# Supplementary material for: Learning curve and surgical outcome of robotic assisted colorectal surgery with ERAS program
Source: Sci Rep. 2022 Nov 29;12:20566. doi: 10.1038/s41598-022-24665-w (PMC9709162; doi:10.1038/s41598-022-24665-w)
Supplement: Supplementary file 2 — Supplementary Information 2. [file 41598_2022_24665_MOESM2_ESM.zip › RAL Raw data-Ñ[▒K/082/OP_ñw╝╨░O▒Kñσ.pdf]

正式摘要(電子簽章：000000216863829)

W95 / W95

臺中榮民總醫院

Operation Note

[redacted]

- 手術日期：2021/05/13
- 手術醫師：VS 蔣鋒帆 , CR 楊曙榮 , R 古朋育 , R 劉佳樺
- Anesthesia : GE
- Position : Supine
- Aseptic agents : Soap , Betadine Alcohol , Betadine Aqua

Diagnosis

- Pre-operative diagnosis : A colon tumor.
- Post-operative diagnosis : Ditto

Surgical Indication

- Tumor resection

Operation

- Robot Assisted Right Hemicolectomy

Finding

- 1. One 1x2 cm polyp located at middle A-colon
  2. There was no palpable liver metastasis, no visible peritoneal seeding.
  3. No Drain
  4. Surgiwrap +
  5.Moderate adhision around T-colon, A-meso colon and omentum

Procedure

- Under GE, the patient was put in supine position. 5 troca hole was made, the first was by open method. Pneumoperitonium was created. the robotic endoscope was inserted. Abdominal exploration was done and the findings were mentioned above. The patient position was adjusted to left tilting to let small bowels move to LLQ and the A-colon was well exposured. The ileocolic vessel was identified, dissected and transected by hemolock. The retroperitoneal dissection was done through embryonic plane between meso-right colon and Gerota fascia. The Duodenum and pancrease head was identified and protected. The mobilization of A-colon was done. Midline was enlarged and A-colon was pull out. Resection of ileum and A-colon with side to side anastomosis was done by GIA 80/3.8mmX2. The abdoman was irrigationwas warm N/W and the Surgiwrap left. 4 ports and wound were closed in layers. Throughout the whole course of operation, patient stood quite well. The patient was sent to POR for post-OP care.
- Estimated blood loss: minimum
- 器械數量: 正確
- 紗布數量: 正確

Image

- PAINT
  - abdomen

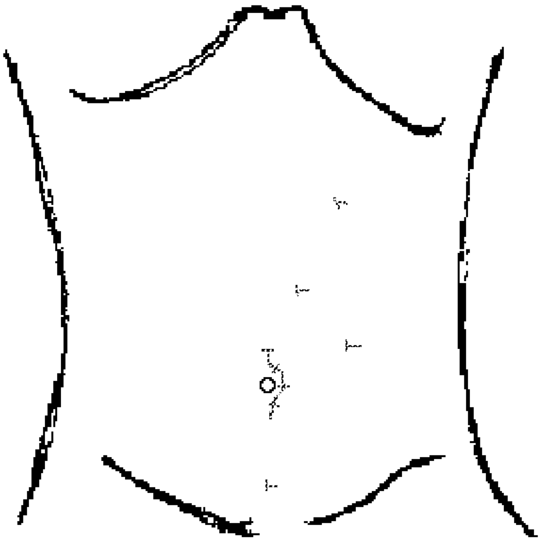

- colon & rectum

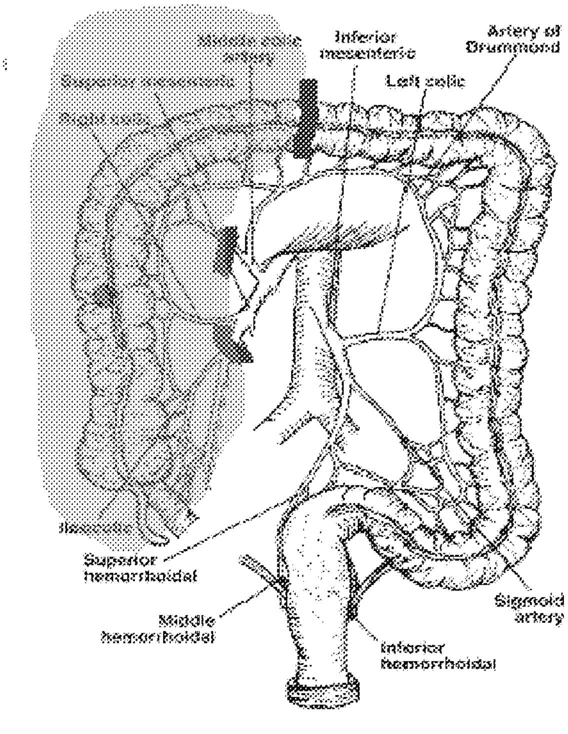

主任 2062F蔣鋒帆 / 醫師 2292G楊曙榮

正式摘要(**電子簽章：000000216863829)**

W95 / W95

doctorRank:B
